# Factors Associated With Mortality Among Hospitalized Children With Acute Bacterial Meningitis in a Resource-Limited Setting: A Retrospective Study

**DOI:** 10.7759/cureus.74827

**Published:** 2024-11-30

**Authors:** Nabil Aljuma'ai, Faisal Ahmed, Mohammed Almogahed, Hanan Al-Barahi, Abdulghani Al-Hagri, Ola Alnadhary, Abdulghani A Ghabisha

**Affiliations:** 1 Department of Pediatrics, Ibb University, Ibb, YEM; 2 Department of Urology, Ibb University, Ibb, YEM; 3 Department of Internal Medicine, School of Medicine, Ibb University, Ibb, YEM; 4 Department of Laboratory Medicine, Al-Thora General Hospital, Ibb, YEM; 5 Student Research Committee, Faculty of Medicine and Health Sciences, Sana'a University, Sana'a, YEM; 6 Department of Pediatrics, School of Medicine, Ibb University, Ibb, YEM

**Keywords:** acute bacterial meningitis, children, ibb, mortality, resource-limited setting, yemen

## Abstract

Background: Acute bacterial meningitis (ABM) is a significant public health problem in developing countries, including Yemen, especially during warfare. This is because persistent political turmoil impedes ABM prevalence, etiology, and treatment. Here, we investigate the factors associated with mortality among hospitalized children with ABM in a resource-limited setting.

Material and method: A retrospective study between March 2018 and December 2023 at the Pediatric Center, Althora General Hospital, Yemen, included 387 children (aged <15 years) diagnosed with ABM and confirmed by bacteriology. The data on patient characteristics and cerebrospinal fluid (CSF) characteristics, culture, and treatment outcome were collected from the patient medical profile and analyzed. Factors associated with mortality were investigated in univariate and multivariate analysis using odds ratio (OR) and 95% confidence interval (CI).

Result: The median age was 1.00 years (interquartile range (IQR): 1.00, 4.00), with most being under one year old (n=213, 55.0%), male (n=237, 61.2%), and from rural areas (n=218, 56.3%), with symptoms lasting over five days in 58 (15%) of cases. Streptococcus pneumonia was the most common CSF culture result (n=383, 99%), with mortality reported in 15 (3.9%) cases. In multivariate analysis, younger age (OR: 1.77; 95% CI: 1.18-2.94, p=0.010), malnutrition (OR: 480.82; 95% CI: 27.78-56020.49, p=0.001), altered mental status (OR: 1536.83; 95% CI: 42.82-658,144.96, p=0.002), and the longer time before hospitalization > five days (OR: 161.84; 95 % CI: 9.97-16,700.53, p=0.005) were associated with mortality.

Conclusion: Our findings highlight the prognostic significance of early detection of a predisposing focus to ABM. Poor prognosis and mortality may be associated with younger age, delayed hospitalization, malnutrition, and altered mental status.

## Introduction

Meningitis, an infection of the central nervous system, poses a significant global health challenge, with the WHO estimating 700,000 cases in 2023, predominantly affecting populations in Africa and Southeast Asia [1]. It impacts 1.2 million individuals annually, with prevalence influenced by age, season, geography, and socioeconomic factors [2]. Acute meningitis arises mainly from bacterial or viral pathogens, while chronic forms are typically due to TB or fungal infections [3,4]. Despite the availability of effective treatments, meningitis results in substantial global morbidity and mortality, with ABM being the most common form, particularly in children under five, accounting for a notable percentage of fatalities [5,6].

Conjugate vaccines have markedly diminished the incidence of community-acquired bacterial meningitis over the past 30 years, prompting multiple organizations to provide financial support for the implementation of pneumococcal, Haemophilus influenza type b (Hib), and meningococcal conjugate vaccinations in economically disadvantaged nations [3,7]. Yemen, a country classified as developing on the Arabian Peninsula, is characterized by a tropical desert along its coastal areas and lowlands, accompanied by mild to moderate precipitation in the western plateau region. The dry season is observed from November to February. The Global Alliance for Vaccines and Immunisation (GAVI) introduced the Hib vaccine in 2005, administering it at ages 6, 10, and 14 weeks. The conjugate pneumococcal vaccine (PCV13) was incorporated into the national immunization program in 2011 [8].

Nevertheless, the civil strife in Yemen has led to a significant rise in suspected cases of acute bacterial meningitis (ABM) among hospitalized pediatric patients, with Streptococcus pneumoniae, Neisseria meningitidis, and H. influenzae type b identified as the predominant pathogens [9]. The ongoing conflict has also adversely affected vaccine coverage, with the Ta'izz region exhibiting the highest rates of suspected ABM alongside the lowest immunization rates [3]. According to this review, the prevalence of alleged ABM in the IBB Governorate was calculated to be 59.6 per 100,000, with a confirmed ABM rate of 21.2% [3]. A previous investigation in northern Yemen estimated that the incidence of ABM was 27.1 per 100,000 for children under five years of age and 84.6 per 100,000 for children under one year of age [9]. In another report, Al Khorasani et al. [10] conducted a study between May 1999 and June 2001 to investigate the bacterial profiles and clinical outcomes of childhood meningitis in Yemen, explicitly focusing on the northern Yemeni population. Their findings indicated that the primary pathogens responsible for childhood ABM were S. pneumoniae and Neisseria meningitidis. Additionally, they noted that meningitis caused by S. pneumoniae was associated with higher rates of mortality and permanent disability [10]. However, research concerning the prevalence, etiological factors, and treatment efficacy of ABM in Yemen requires further development due to the persistent civil unrest, necessitating additional studies to enhance understanding of the ABM epidemic and the effectiveness of vaccination strategies.

This study aims to explore the prevalence and mortality determinants of ABM among hospitalized children within a resource-constrained environment at Althora University Hospital in Ibb, Yemen.

## Materials and methods

Study design and setting

A retrospective cross-sectional study was carried out at the Pediatric Center, Althora General Hospital, Ibb, Yemen, between March 2018 and December 2023, including 387 children diagnosed with ABM and confirmed by bacteriology. The IBB city is located in southern Yemen, the seat of Ibb Governorate, around 117 km (73 miles) northeast of Mocha and 194 km (121 miles) south of Sanaa. Al-Thora General Hospital is our city's sole governmental hospital offering pediatric specialty services. The hospital currently serves more than two million people. Furthermore, since 2015, it has covered more patients, including the Taiz Governate and other governments that ceased or did not function due to restrictive policies during the current conflicts [11].

Inclusion Criteria

Children under 15 with ABM confirmed through positive bacteriological investigation, including direct detection of bacterial infections through CSF fluid and/or culture. The real-time polymerase chain reaction (PCR) was unavailable in our laboratory and not included in the diagnosis.

Exclusion Criteria

Patients aged > 15 years, children with meningitis caused by various agents (viruses, fungi, TB, parasites, etc.) who are culture-negative, and those treated in other centers were excluded.

Data collection

A pre-established case investigation form was used to collect data, supplemented by clinical information from patient records and laboratory results. The investigator meticulously gathered data through independent chart reviews. The data abstraction tool was developed after an extensive review of relevant literature on pediatric bacterial meningitis [3,5,10,12,13].

The collected data underwent thorough assessment for accuracy, completeness, and consistency. Data gatherers participated in training sessions to ensure reliability, and the checklist was pre-validated. Regular reviews were conducted to ensure data consistency and completeness. The supervisor and the principal investigator reviewed all collected data to verify completeness and consistency. If any missing information was identified, corrective actions were taken. Any contradictory findings or those needing more details prompted subsequent review and reevaluation of the charts.

Established standard operating procedures were meticulously adhered to during the laboratory analysis of cerebrospinal fluid (CSF) cultures. To ensure the integrity of the results, any CSF samples that exhibited gross contamination with blood were not received within two hours post-collection or were not maintained in an appropriate transport medium and were systematically discarded. Culture media were formulated and sterilized following the prescribed standard operating procedures. The sterility of the culture media was verified through the parallel incubation of un-inoculated plates alongside each batch of newly prepared culture plates, with careful observation for any bacterial proliferation. Control strains, specifically S. pneumoniae ATCC 49619 and E. coli ATCC 25922, procured from the Yemenin Public Health Institute, were employed as quality controls during CSF cultures, biochemical assays, and antimicrobial susceptibility assessments.

The variables studied included age (divided into < 1 year, 1-5 years, 6-10 years, and 11-15 years), gender, residency status (rural vs. urban), season of admission (winter vs. summer), nutritional status (malnourished vs. normal), Hib vaccination status, time between the onset of symptoms and hospitalization (≤ 5 days vs. > 5 days), clinical symptoms at presentation (fever, vomiting, bulging fontanelle, altered mental status, headache, stiff neck, skin rash, and local paralysis), history of antibiotic use in the week prior, results of CSF bacteriological examination (glucose, white blood cell count, and protein), CSF culture results, duration of hospital stay (≤ 5 days, 5-14 days, and > 14 days), and treatment outcomes (living vs. deceased).

This study defined ABM as the sudden onset of clinical symptoms such as fever, headache, seizure, vomiting, and decreased altered mental status within four weeks after infection and a positive bacterial CSF culture [6,14]. Finally, collected data were verified for accuracy, cleaned, entered into Epi-Data (version 4.6; https://www.epidata.dk), and analyzed using Statistical Product and Service Solutions (SPSS, version 25; IBM SPSS Statistics for Windows, Armonk, NY).

Primary outcome

The primary outcome pertains to the prevalence of ABM among admitted children, wherein the pathogen serves as a causative agent for ABM and its associated mortality alongside its predictive determinants.

Statistical analysis

The data were extensively reviewed for completeness and consistency, cleansed, and loaded into SPSS software for analysis. We conducted a descriptive analysis of the entire sample, reporting quantitative data as mean ± standard deviation or median and interquartile range (IQR) and qualitative variables as frequencies and percentages. Binary logistic regression analysis was performed, and candidate variables were chosen at a p-value of < 0.05. Multiple logistic regression was used, and independent predictors of acute bacterial meningitis death were discovered at a p-value of < 0.05, along with odds ratios (OR) and 95% confidence intervals (CI). The survival rate was calculated using Kaplan-Meier curves, and log-rank tests were used to compare survival curves based on risk variables.

Ethical approval

The study was conducted following the Declaration of Helsinki and approved by the Ibb University Institutional Ethics Committee (Code: IBBUNI.AC.YEM. 2023.104. 82 on 2023-06-13). Due to the anonymous retrospective nature of the study, written informed consent from the included patients was not required.

## Results

Participants' demographic and baseline characteristics

The median age was 1.00 years (IQR: 1.00, 4.00) with a mean of 2.97±3.10 years (range: 1-15 years), and most cases (n=213, 55.0%) were less than one year. One hundred and seven (27.6%) were aged between one and five years, and 67 (17.3%) were older than five years. Most cases were male (n=237, 61.2%) and from rural areas (n=218, 56.3%). The duration between symptom presentation and hospital admission was more than five days in 54 (15.0%). There were 65 (16.8%) malnourished children. Most meningitis cases occurred in winter 343 (88.6%). Symptoms and physical examination findings during admission were fever (363, 93.8%), vomiting (207, 53.5%), headache (19, 4.9%), neck stiff (11, 2.8%), seizures (99, 25.6%), altered mental status (116, 30.0%), bulging fontanelle (12, 3.1%), skin rash (6, 1.6%), and local paralysis (2, 0.5%). The majority of cases (n=290, 74.9) received complete vaccinations, while 97 (25.1) reported incomplete vaccination. Most cases (95.6%) had a history of previous antibiotic treatment one week before admission (Table 1).

**Table 1:** Demographic characteristics of children patients with acute bacterial meningitis and univariate analysis for factors associated with mortality. Abbreviations: IQR: interquartile range, NA: not applicable, OR: odds ratio, CI: confidence interval Notes: The analysis was made via ^1^linear model ANOVA and ^2^Pearson's chi-squared test. A p-value less than 0.05 was considered statistically significant.

Variables	Subgroups	Total (n=387)	Alive (N=372, 96.1%)	Died (N=15, 3.9%)	OR (95% CI)	p-value
Age (year)	Median (IQR)	1.0 (1.0 to 4.0)	3.0 (2.0 to 6.5)	1.0 (1.0 to 4.0)	1.15 (1.00-1.31)	0.025^1^
Gender	Male	237 (61.2)	225 (60.5)	12 (80.0)	Reference group	0.128^2^
	Female	150 (38.8)	147 (39.5)	3 (20.0)	0.38 (0.09-1.23)	
Nutritional status	Normal	322 (83.2)	318 (85.5)	4 (26.7)	Reference group	< 0.001^2^
	Malnourished	65 (16.8)	54 (14.5)	11 (73.3)	16.19 (5.33-60.14)	
Residency	Urban	169 (43.7)	166 (44.6)	3 (20.0)	Reference group	0.059^2^
	Rural	218 (56.3)	206 (55.4)	12 (80.0)	3.22 (1.00-14.32)	
Season	Winter	343 (88.6)	333 (89.5)	10 (66.7)	Reference group	0.006^2^
	Summer	44 (11.4)	39 (10.5)	5 (33.3)	4.27 (1.28-12.68)	
Temperature	Afebrile	24 (6.2)	23 (6.2)	1 (6.7)	Reference group	0.939^2^
	Febrile (> 38℃)	363 (93.8)	349 (93.8)	14 (93.3)	0.92 (0.17-17.08)	
Seizure	No	288 (74.4)	282 (75.8)	6 (40.0)	Reference group	0.002^2^
	Yes	99 (25.6)	90 (24.2)	9 (60.0)	4.70 (1.65-14.36)	
Vomiting	No	180 (46.5)	170 (45.7)	10 (66.7)	Reference group	0.110^2^
	Yes	207 (53.5)	202 (54.3)	5 (33.3)	0.42 (0.13-1.21)	
Bulging fontanelle	No	375 (96.9)	360 (96.8)	15 (100.0)	Reference group	0.480^2^
	Yes	12 (3.1)	12 (3.2)	0 (0.0)	NA	
Altered mental status	Not change	271 (70.0)	268 (72.0)	3 (20.0)	Reference group	< 0.001^2^
	Decreased	116 (30.0)	104 (28.0)	12 (80.0)	10.52 (3.25-47.01)	
Headache	No	368 (95.1)	354 (95.2)	14 (93.3)	Reference group	0.748^2^
	Yes	19 (4.9)	18 (4.8)	1 (6.7)	1.40 (0.08-7.61)	
Stiff neck	No	376 (97.2)	361 (97.0)	15 (100.0)	Reference group	0.499^2^
	Yes	11 (2.8)	11 (3.0)	0 (0.0)	NA	
Skin rash	No	381 (98.4)	366 (98.4)	15 (100.0)	Reference group	0.620^2^
	Yes	6 (1.6)	6 (1.6)	0 (0.0)	NA	
Local paralysis	No	385 (99.5)	370 (99.5)	15 (100.0)	Reference group	0.776^2^
	Yes	2 (0.5)	2 (0.5)	0 (0.0)	NA	
Antibiotics week prior	Yes	370 (95.6)	356 (95.7)	14 (93.3)	Reference group	0.661^2^
	No	17 (4.4)	16 (4.3)	1 (6.7)	1.59 (0.09-8.70)	
Vaccination status	Complete	290 (74.9)	277 (74.5)	13 (86.7)	Reference group	0.285^2^
	Incomplete	97 (25.1)	95 (25.5)	2 (13.3)	0.45 (0.07-1.66)	
Duration of symptoms before hospitalization	≤ 5 days	329 (85.0)	321 (86.3)	8 (53.3)	Reference group	< 0.001^2^
	> 5 days	58 (15.0)	51 (13.7)	7 (46.7)	5.51 (1.86-15.99)	

All cases underwent lumbar punctuation, and CSF was cloudy in most cases (379, 97.9%). In most cases, the WBCs were greater than 2.000 per μL (n=331, 85.5%). Polymorphonuclear neutrophil leukocytes (PMNs) were greater than 1,180 (mg/dL); in most cases, they were 381 (98.4%). The glucose was less than 34 (mg/dL) in most cases (n=377, 97.4%), and protein was greater than 220 (mg/dL) in most cases 381 (98.4%). The most common CSF culture result was S. pneumonia (n=384, 99.2%). Other reports were Neisseria meningitides and H. influenza type b in two (0.5%) and one (0.3%), respectively. All the patients received antibiotics (penicillin or ampicillin and cephalosporins), and the duration of hospitalization was less than five days, between five and 14 days, and more than 14 days in 280 (72.4%), 90 (23.3%), and 17 (4.4%), respectively (Table 2).

**Table 2:** Laboratory data and outcome of hospitalized children with acute bacterial meningitis and univariate analysis for factors associated with mortality. Abbreviations: WBC: white blood cell, CSF: cerebrospinal fluid, NA: not applicable, OR: odds ratio, CI: confidence interval Notes: The analysis was made via ^1^linear model ANOVA and ^2^Pearson's chi-squared test. A p-value less than 0.05 was considered statistically significant. *Other germs were Neisseria meningitides and Haemophilus influenza type b.

Variables	Subgroups	Total (N=387)	Alive (N=372, 96.1%)	Died (N=15, 3.9%)	OR (95% CI)	p-value
CSF fluid appearance	Cloudy	379 (97.9)	364 (97.8)	15 (100.0)	Reference group	0.848^1^
	Yellow	5 (1.3)	5 (1.3)	0 (0.0)	NA	
	Pink	3 (0.8)	3 (0.8)	0 (0.0)	NA	
CSF culture	Streptococcus pneumonia	383 (99.0)	370 (99.5)	13 (86.7)	Reference group	< 0.001^1^
	Other*	4 (1.0)	2 (0.5)	2 (13.3)	28.46 (3.22-253.04)	
Hospitalization	Less than 5 days	280 (72.4)	268 (72.0)	12 (80.0)	Reference group	0.616^1^
	Between 5-14 days	90 (23.3)	88 (23.7)	2 (13.3)	0.51 (0.08-1.91)	
	More than 14 days	17 (4.4)	16 (4.3)	1 (6.7)	1.40 (0.07-7.79)	
CSF WBCs	≥2000 per μL	331 (85.5)	322 (86.6)	9 (60.0)	Reference group	0.004^1^
	<2000 per μL	56 (14.5)	50 (13.4)	6 (40.0)	4.29 (1.39-12.43)	
CSF Glucose	≤ 70 (mg/dL)	10 (2.6)	8 (2.2)	2 (13.3)	Reference group	0.053^1^
	> 70 (mg/dL)	377 (97.4)	364 (97.8)	13 (86.7)	0.14 (0.03-1.01)	
CSF Protein	> 100 (mg/dL)	6 (1.6)	6 (1.6)	0 (0.0)	Reference group	0.620^1^
	≤ 100 (mg/dL)	381 (98.4)	366 (98.4)	15 (100.0)	NA	

Survival analysis

**Figure 1:**
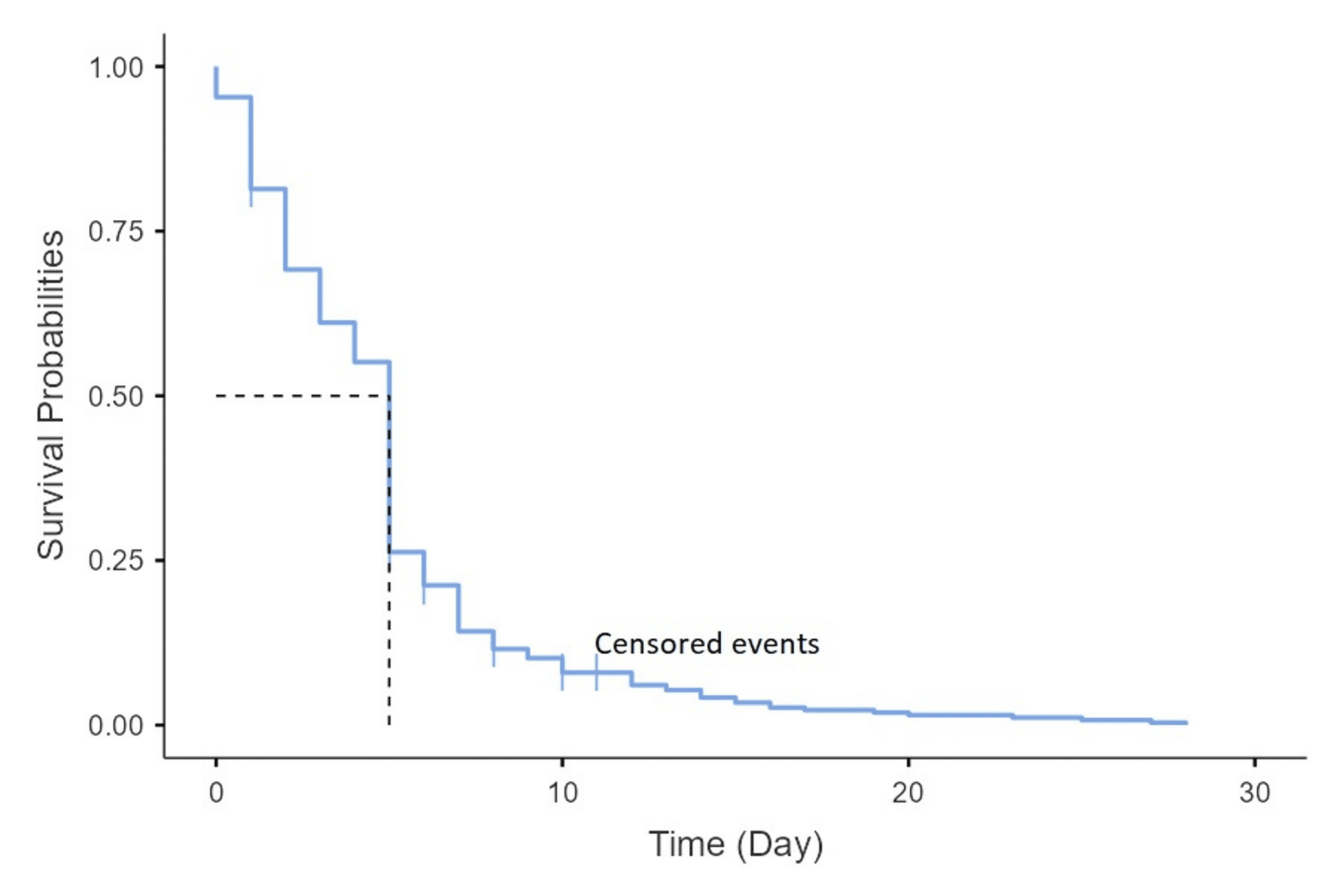
Kaplan-Meier curves for the overall mortality associated with acute bacterial meningitis.

The mortality was reported in 15 (3.9%) cases. The median length of hospitalization was 11 days, with a maximum of 32 days. For death cases, the median duration was 6.5 days, compared to 12 days for living controls. The survival curve gradually decreased, with the most significant mortality occurring in the first five days (12 of 15 died cases) (Figure 1).

Factors associated with mortality

In univariate analysis, younger age (p=0.025), malnutrition (p<0.001), season (p=0.006), the time before hospitalization of more than five days (p<0.001), seizure (p=0.002), altered mental status (p<0.001), lower WBCs in CSF (p=0.004), and pathogenic organisms with non-S. pneumonia etiology (p<0.001) were associated with mortality. However, in multivariate analysis, only younger age (OR: 1.77; 95% CI: 1.18-2.94, p=0.010), malnutrition (OR: 480.82; 95% CI: 27.78-56,020.49, p=0.001), altered mental status (OR: 1536.83; 95% CI: 42.82-658,144.96, p=0.002), and the longer time before hospitalization > five days (OR: 161.84; 95% CI: 9.97-16,700.53, p=0.005) were associated with mortality and were statistically significant (Table 3).

**Table 3:** Multivariate analysis for factors associated with mortality in pediatric patients with acute bacterial meningitis. Abbreviations: SD: standard deviation, WBC: white blood cell, CSF: cerebrospinal fluid, OR: odds ratio, CI: confidence interval Note: A p-value less than 0.05 was considered statistically significant. *Other germs are Neisseria meningitides and Haemophilus influenza type b.

Variables	Subgroups	Alive (N=372, 96.1%)	Died (N=15, 3.9%)	OR (95% CI)	p-value
Age (year)	Mean ± SD	4.7 ± 4.2	2.9 ± 3.0	1.77 (1.18-2.94)	0.010
Nutritional status	Normal	318 (98.8)	4 (1.2)	Reference group	0.001
	Malnourished	54 (83.1)	11 (16.9)	480.82 (27.78-56020.49)	
Season	Winter	333 (97.1)	10 (2.9)	Reference group	0.241
	Summer	39 (88.6)	5 (11.4)	5.43 (0.38-166.37)	
Seizure	No	282 (97.9)	6 (2.1)	Reference group	0.246
	Yes	90 (90.9)	9 (9.1)	3.31 (0.42-26.74)	
Altered mental status	Not change	268 (98.9)	3 (1.1)	Reference group	0.002
	Decreased	104 (89.7)	12 (10.3)	1536.83 (42.82-658144.96)	
Duration of symptoms before hospitalization	≤ 5 days	321 (97.6)	8 (2.4)	Reference group	0.005
	> 5 days	51 (87.9)	7 (12.1)	161.84 (9.97-16700.53)	
CSF culture	Streptococcus pneumonia	370 (96.6)	13 (3.4)	-	0.150
	Other*	2 (50.0)	2 (50.0)	136.38 (0.66-127894.04)	
CSF WBCs	≥2000 per μL	322 (97.3)	9 (2.7)	-	0.655
	<2000 per μL	50 (89.3)	6 (10.7)	1.63 (0.15-12.91)	

## Discussion

Bacterial meningitis has historically been a leading cause of pediatric morbidity and mortality worldwide, especially in low- and middle-income nations [15]. This study examined acute bacterial meningitis's clinical and laboratory characteristics and predictive factors in 387 pediatric patients. Our findings indicated that S. pneumoniae was the predominant pathogen, with a mortality rate of 3.9%. Additionally, factors such as younger age, malnutrition, altered mental status, and delayed presentation exceeding five days were significant mortality determinants in multivariate analysis.

In this study, S. pneumoniae emerged as the most prevalent pathogen. Our results align with prior studies conducted in Yemen from 1999 to 2001 and from 2014 to 2020, as well as those in Vietnam (2019-2021), Madagascar (2012-2015), and China (2019-2020) [3,5,10,12,13]. Furthermore, while the pathogenic etiology of non-S. pneumoniae meningitis correlated with mortality, it lacked statistical significance in multivariate analysis due to limited sample size. A recent systematic review encompassing 13,082 studies demonstrated a robust association between pneumococcal meningitis and mortality, with a pooled case fatality rate of 24.59%, predominantly affecting children under five years [16]. Additionally, recent findings by Pelkonen et al. established a connection between ABM severity indicators and non-meningococcal etiology [17].

Our study's mortality rate of 3.9% was lower than that reported in other developing countries, such as Nguyen-Huu et al. [5] (12.1% in Vietnam) and Al Khorasani et al. [10] (10% in Yemen). However, it was higher than the findings from Mioramalala et al. [12] (0.6%) and Sadeq et al. [18] (2.5%). In another study from Ethiopia, Tewabe et al. found that about 15% of children with meningitis developed poor outcomes [19]. In another report from Child Health and Mortality Prevention Surveillance (CHAMPS), catchments in six sub-Saharan African countries (Ethiopia, Kenya, Mali, Mozambique, Sierra Leone, South Africa) and Bangladesh were conducted between 2016 and 2023. Mahtab et al. revealed that meningitis contributed to 7.0% of under five deaths [20]. The lower mortality rate in this study may be influenced by the relatively small sample size, resulting from the exclusion of children with incomplete data or those lacking provisional ABM diagnosis via CSF culture. Additionally, our single-center retrospective study cannot accurately represent the mortality rate due to ABM in Yemen. The absence of pediatric intensive care unit (ICU) facilities may also have contributed to lower mortality rates as many critically ill patients were referred to other tertiary centers. Therefore, our findings necessitate careful interpretation.

The incidence of ABM cases demonstrates significant age-related variability. In this study, the highest prevalence of ABM was observed in children under one year (55.0%), while the lowest occurred in those over five years (17.3%). Our results corroborate previous studies conducted in Yemen and other developing nations such as Oman, Kuwait, and Sudan [3,7,10,18,21]. Furthermore, we identified younger age as a predictor of mortality in children with ABM. In a similar study, Mioramalala et al. reported that infants under six months with bacterial meningitis face double the mortality risk compared to older children [12]. The low ABM prevalence among older children was attributed to the immune system maturity in older children [5]. Furthermore, in this study, a male predominance in ABM cases is consistently observed across demographics, although gender does not affect mortality rates. Our findings corroborate earlier research indicating higher male incidence in pediatric ABM, with varying ratios internationally [12,22,23]. The study reveals a higher prevalence of ABM in rural areas and in summer, aligning with previous research [5,14]. However, these factors do not predict mortality due to sample size limitations. Additionally, prior studies show inconsistencies in ABM prevalence concerning rurality and seasonal variations, with winter admissions exhibiting lower mortality due to infrastructural challenges in the rainy season [24].

Malnourished patients exhibited increased mortality rates compared to adequately nourished individuals, a finding consistent with studies from Ethiopia and Yemen [14,25]. This is attributed to the compromised immune systems of malnourished children, which exacerbate infections and complicate treatment, emphasizing the critical role of nutrition in ABM outcomes.

In our report, fever and vomiting were the predominant presenting symptoms, aligning with previous studies [5,7]. However, symptomatology exhibits considerable age-related variability, with younger children displaying more nonspecific symptoms [26]. Furthermore, we established a correlation between severe neurological symptoms and increased ABM mortality, corroborating earlier findings [14,27,28]. Additionally, a systematic review highlighted a strong association between prolonged seizures and mortality risk [29].

This study determined that delayed hospitalization (beyond five days from symptom onset) correlates with heightened mortality, consistent with findings from various global studies [14,27,29,30]. It is well-documented that late presentation with advanced clinical features, particularly in suboptimal critical care settings, results in increased mortality rates due to therapeutic challenges [14].

In this investigation, a CSF WBC count of 2,000 per μL or less indicated poor outcomes in univariate analysis, although it lacked statistical significance in multivariate analysis. Similarly, Lin et al. identified a relationship between lower CSF WBC counts and adverse outcomes. The authors indicated low CSF WBC and glucose levels are linked to elevated mortality risk in pneumococcal meningitis cases [31]. Potential explanations involve heightened bacterial activity and severe inflammation, though further research is required to elucidate these connections.

Study limitations

Study limitations include reliance on secondary data, which may exhibit variability in quality due to documentation practices. The retrospective design may introduce intrinsic biases, and excluding inadequate records could lead to selection bias. Furthermore, essential factors influencing pediatric ABM mortality, such as family dynamics and socioeconomic status, were not documented. Our findings provide valuable insights into potential mortality prognostic indicators in pediatric ABM cases. Our study's limitations also include the absence of PCR testing. Additionally, this study was conducted in an educational hospital in Yemen; generalizability to other contexts may be limited. We recommend prospective trials with larger sample sizes, multicentric, and extended postoperative follow-up to enhance result robustness.

## Conclusions

This investigation examined the various factors contributing to fatalities from pediatric ABM in Yemen to improve patient care and reduce mortality risks. Our findings indicated that S. pneumoniae was the most common pathogen, with a mortality rate of 3.9%. Additionally, factors such as younger age, malnutrition, altered mental status, and delayed presentation (beyond five days) were identified as significant determinants of mortality in our multivariate analysis. These factors associated with ABM-related mortality align with those previously documented in existing literature.

Identifying at-risk patients is crucial for providing appropriate support during admission. Further research is needed to address the current study's limitations, particularly in developing a pediatric ABM severity score and evaluating emergency care, which is extremely important. Furthermore, comprehensive neurological assessments, thorough diagnostic laboratory investigations, and prompt antibiotic administration are essential to minimize adverse outcomes.
